# Making Quality Health Websites a National Public Health Priority: Toward Quality Standards

**DOI:** 10.2196/jmir.5999

**Published:** 2016-08-02

**Authors:** Theresa Devine, Jordan Broderick, Linda M Harris, Huijuan Wu, Sandra Williams Hilfiker

**Affiliations:** ^1^ U.S. Department of Health and Human Services (HHS), Office of Disease Prevention and Promotion (ODPHP) Rockville, MD United States; ^2^ U.S. Department of Health and Human Services (HHS), National Vaccine Program Office Washington, DC United States; ^3^ CommunicateHealth, Inc. Northampton, MA United States

**Keywords:** online health information, health literacy, reliability, usability, measurement

## Abstract

**Background:**

Most US adults have limited health literacy skills. They struggle to understand complex health information and services and to make informed health decisions. The Internet has quickly become one of the most popular places for people to search for information about their health, thereby making access to quality information on the Web a priority. However, there are no standardized criteria for evaluating Web-based health information. Every 10 years, the US Department of Health and Human Services' Office of Disease Prevention and Health Promotion (ODPHP) develops a set of measurable objectives for improving the health of the nation over the coming decade, known as Healthy People. There are two objectives in Healthy People 2020 related to website quality. The first is objective Health Communication and Health Information Technology (HC/HIT) 8.1: increase the proportion of health-related websites that meet 3 or more evaluation criteria for disclosing information that can be used to assess information reliability. The second is objective HC/HIT-8.2: increase the proportion of health-related websites that follow established usability principles.

**Objective:**

The ODPHP conducted a nationwide assessment of the quality of Web-based health information using the Healthy People 2020 objectives. The ODPHP aimed to establish (1) a standardized approach to defining and measuring the quality of health websites; (2) benchmarks for measurement; (3) baseline data points to capture the current status of website quality; and (4) targets to drive improvement.

**Methods:**

The ODPHP developed the National Quality Health Website Survey instrument to assess the quality of health-related websites. The ODPHP used this survey to review 100 top-ranked health-related websites in order to set baseline data points for these two objectives. The ODPHP then set targets to drive improvement by 2020.

**Results:**

This study reviewed 100 health-related websites. For objective HC/HIT-8.1, a total of 58 out of 100 (58.0%) websites met 3 or more out of 6 reliability criteria. For objective HC/HIT-8.2, a total of 42 out of 100 (42.0%) websites followed 10 or more out of 19 established usability principles. On the basis of these baseline data points, ODPHP set targets for the year 2020 that meet the minimal statistical significance—increasing objective HC/HIT-8.1 data point to 70.5% and objective HC/HIT-8.2 data point to 55.7%.

**Conclusions:**

This research is a critical first step in evaluating the quality of Web-based health information. The criteria proposed by ODPHP provide methods to assess website quality for professionals designing, developing, and managing health-related websites. The criteria, baseline data, and targets are valuable tools for driving quality improvement.

## Introduction

Most US adults (90%) have limited health literacy skills [1], which means that many struggle to make sense of the complex information and services intended to help prevent disease and promote our health [2-5]. One promising path to improve the health literacy of the adult population is to increase the availability of evidence-based, understandable, easy-to-find sources of health information [6-8].

While medical experts continue to play a vital role in the health decision–making process, the Internet has quickly become one of the most popular places for people to search for information about their health [9,10]. Research indicates that not only are adults of all generations searching online for health information [11,12], but they are using what they find to make health care decisions, either on behalf of themselves or a loved one [13]. Thus, improving the quality of health-related websites has the potential to improve the health literacy—and the health—of the population [14,15].

Increasing access to quality online information is a shared priority among national and international policy makers. President Obama’s Digital Government Strategy calls for new and better ways to deliver digital information and services [16] and the World Health Organization has called for greater transparency, privacy and security, codes of conduct, and individual choice and control of health-related websites [17].

Yet, in spite of the critical role of quality in health-related websites, there are no standardized criteria for assessing it [6,8,18]. Such criteria have been elusive since researchers called for operationalized definitions of quality in their 2002 meta-analysis of empirical website assessments [7]. Therefore, the first steps in achieving real gains in health-related website quality are to establish (1) a standardized approach to defining and measuring the quality of health websites; (2) benchmarks for measurement; (3) baseline data points to capture the current status of website quality; and (4) targets to drive improvement. This paper describes the efforts of the US Department of Health and Human Services’ Office of Disease Prevention and Health Promotion (ODPHP) to establish these four items.

## Methods

### Defining and Measuring Quality Health Websites for Healthy People

Every 10 years, ODPHP manages the development of a set of measurable objectives for improving the health of the nation over the coming decade, known as Healthy People. Two objectives (Health Communication and Health Information Technology, HC/HIT-8.1 and 8.2, see Textbox 1) in Healthy People 2020 relate to website quality: one calls for improved information reliability; the other calls for improved website usability. Both were devised and operationalized by experts in health communication and technology. Together, these objectives provide a working definition of website quality and a promising path toward overall quality improvement of Web-based health information.

**Healthy People objectives Health Communication and Health Information Technology (HC/HIT) 8.1 and 8.2.**

HC/HIT-8.1: Increase the proportion of health-related websites that meet three or more evaluation criteria for disclosing information that can be used to assess information reliability

HC/HIT-8.2: Increase the proportion of health-related websites that follow established usability principles

### Information Reliability

Information reliability refers to the accuracy and credibility of website content as well as transparency in the purpose and ownership of the site [7,19]. This information can help users discern the origin and quality of Web-based content [20]. It is one of the most commonly identified indicators of website quality and has been widely referenced by public, private, and nonprofit organizations committed to improving the quality of Web-based health information such as the Medical Library Association, Health on the Net Foundation, Consumers Union, and the Agency for Healthcare Research and Quality [21-24].

Healthy People objective HC/HIT-8.1 was first introduced in Healthy People 2010 as a developmental objective. In 2005, ODPHP convened a Technical Expert Workgroup to identify reliability criteria based on established Web standards [25,26]. The ODPHP then developed the Website Information Reliability Evaluation Instrument (Multimedia Appendix 1), which includes specific reliability requirements for each of the 6 criteria identified by the Workgroup (see Table 1).

**Table 1:** Objective HC/HIT-8.1 criteria and reliability requirements.

Criteria	Reliability requirements
Identity	Name of person or organization responsible for website Street address for person or organization responsible for website Identified source of funding for website
Purpose	Statement of purpose or mission for website Uses and limitations of services provided Association with commercial products or services
Content development	Differentiation of advertising from nonadvertising content Medical, editorial, or quality review practices or policies Authorship of health content (per page of health content)
Privacy	Privacy policy How personal information is protected
User feedback	Feedback form or mechanism How information from users is used^a^
Content updating	Date content created (per page of health content) Date content reviewed, updated, modified, or revised (per page of health content) Copyright date^a^

^a^ Optional requirements.

### Website Usability

Usability standards tend to fall into three major categories [27]. The first category focuses on how the information is organized, commonly referred to as *information architecture*. The second category looks at how users navigate the information on a website, known as *site design*. The third category emphasizes how users interact with content on the website, referred to as *content design*. These three elements are commonly addressed in both federal and private guidelines on website usability [27-32].

Usability is an important component of website quality, affecting a user’s ability to access and understand information online [33]. In fact, the design of a website is one of the most important indicators of website credibility and quality for users [34-37].

In 2012, an expert panel was used to establish an empirical definition of usability for Healthy People 2020. Panel members were selected from academic, private, and government sectors based on their expertise in website usability and health communication. With input from the panel, ODPHP developed the Website Usability Evaluation Instrument (see Multimedia Appendix 2) to measure progress toward Healthy People objective HC/HIT-8.2. The instrument assesses the three aforementioned website usability categories, using 19 established usability principles across 59 task-based usability measures (see Table 2). The Site Design category includes 9 composites that assess basic design elements of the site, including how the site looks, how the site functions, and how a user can interact with the site. The Information Architecture category includes 7 composites that assess how the site content is organized, including navigation, grouping, and labeling. Lastly, the Content Design category includes 3 composites that assess how the content is written and formatted, and includes plain language principles. Each of the 59 measures is rated on a scale of 1 to 4 based on the level of difficulty of performing the task on the website (1 being “task failure” and 4 being “minimal problems”). An average rating score is calculated for each usability principle. The benchmark was set to require an average score of 3.5 for 10 or more of the 19 usability principles.

**Table 2:** Objective HC/HIT-8.2 established usability principles and measures.

Categories	Established usability principles
Site Design	1. Use conventional interaction elements 2. Make it obvious what is clickable and what is not 3. Minimize vertical scrolling 4. Ensure that the Back button behaves predictably 5. Provide clear feedback signals for actions 6. Ensure site is accessible for users with disabilities and uses elements of 508 compliance 7. Provide a simplified user experience 8. Incorporate multimedia 9. Offer a functional home page
Information Architecture	10. Present a clear visual hierarchy 11. Provide easy search functionality 12. Clearly label content categories 13. Make pages easy to skim or scan 14. Make elements on the page easy to read 15. Visually group related topics 16. Make sure text and background colors contrast
Content Design	17. Focus the writing on audience and purpose 18. Use the users’ language; minimize jargon and technical terms 19. Allow for interaction with the content

With Healthy People objectives HC/HIT-8.1 and 8.2, ODPHP established standardized criteria to define and measure the quality of health-related websites and set benchmarks for measurement. Next, the criteria were applied to a sample of health-related websites in order to identify baseline data points, which are required for all Healthy People measurable objectives. The reliability (objective HC/HIT-8.1) and usability (objective HC/HIT-8.2) instruments were combined into a single instrument: the National Quality Health Website Survey. Targets were set to drive improvement by the year 2020.

### Sampling

The ODPHP identified the 100 top-ranked health-related websites (see Multimedia Appendix 3) for 3 months (August-October 2014) from the Alexa Top Sites pool—health category [38]. The data were collected from Alexa on October 14, 2014. Alexa’s traffic ranks are based on the traffic data provided by users in Alexa’s global data panel over a rolling 3-month period. A site’s ranking is based on a combined measure of unique visitors and page views [39]. Websites were considered health related if they had at least three items of health information as it is broadly defined by the e-Health Code of Ethics (see Textbox 2) [40]. Duplicated websites were consolidated with the exception of microsites within the National Institutes of Health such as PubMed and MedlinePlus. A number of websites were excluded from the sample based on the exclusion criteria in Textbox 3.

**Criteria for selecting health websites (from e-Health Code of Ethics).**

- Health information includes information for staying well, preventing and managing disease, and making other decisions related to health and health care.
- It includes information for making decisions about health products and health services.
- It may be in the form of data, text, audio, and/or video.
- It may involve enhancements through programming and interactivity.
- Health products include drugs, medical devices, and other goods used to diagnose and treat illnesses or injuries or to maintain health. Health products include both drugs and medical devices subject to regulatory approval by agencies such as the US Food and Drug Administration or UK Medicines Control Agency and vitamin, herbal, or other nutritional supplements and other products not subject to such regulatory oversight.
- Health services include specific, personal medical care or advice; management of medical records; communication between health care providers and/or patients and health plans or insurers or health care facilities regarding treatment decisions, claims, billing for services, and so on; and other services provided to support health care.
- Health services also include listservs, bulletin boards, chat rooms, and other online venues for the exchange of health information.
- Like health information, health services may be in the form of data, text, audio, and/or video and may involve enhancements through programming and interactivity.

**Exclusion criteria.**

- Sites that are not about human beings
- Sites that are owned or maintained in a foreign country
- Sites that are specifically for health industry professional development, listing job postings for health professionals or research grants available for health researchers
- Sites that are designed only to introduce, sell, or support specific medical commercial products or technology solutions for the health or medical industry
- Sites that provide platforms for laboratory services
- Sites accessible only to members or paying subscribers who must enter an identifying log-in name and password
- Sites about beauty or cosmetic products or hairstyles
- Sites about health or medical education programs
- Sites about fitness industry professional development or gym memberships
- Sites providing pharmacy price comparison information
- Online forums or groups, or other social media platforms for informal discussions regarding health

The final sample included websites sponsored by three types of organizations: 48 out of 100 websites (48.0%) were for profit, 36 out of 100 (36.0%) were nonprofit, and 16 out of 100 (16.0%) were government.

### Interrater Reliability

A senior usability researcher and a research associate reviewed the sample websites, following a reviewer training process to reach a certain level of interrater reliability (IRR). The team used Altman’s Kappa Benchmark Scale (see Table 3) [41].

**Table 3:** Altman’s Kappa Benchmark Scale.

Kappa statistic	Strength of agreement
<.20	Poor
.21-.40	Fair
.41-.60	Moderate
.61-.80	Good
.81-1.00	Very good

Objective HC/HIT-8.1 criteria are primarily composed of yes or no questions. Cohen’s kappa was used to measure the agreement for nominal scales. A benchmark kappa score of .80 (a score generally accepted as demonstrating a sufficient degree of IRR) was used for objective HC/HIT-8.1 [41]. The criteria for objective HC/HIT-8.2 are scored on a 4-point scale, making it more difficult to reach a perfect IRR. A benchmark kappa score of .61 was used for objective HC/HIT-8.2. Interclass correlation (ICC) was used for assessing ordinal and interval scales. To ensure IRR, both reviewers assessed the same 6 websites during the initial training process. The IRR was calculated for each assessment; discrepancies were identified and resolved between the 2 reviewers until the IRR scores met the benchmark kappa scores.

After the initial training process, the reviewers randomly selected 4 additional websites from the sample to measure IRR scores. The IRR scores for the Website Information Reliability Evaluation Instrument (kappa .83) and the Website Usability Evaluation Instrument (ICC .76) both met the benchmarks.

### Website Review Process

After ensuring IRR, the reviewers divided the remaining 90 websites into 2 equal groups. Each reviewer assessed an equal number of websites.

To score each item in the Website Information Reliability Evaluation Instrument, reviewers began by randomly selecting 3 pages from different sections of the site. Each of the items in the instrument was scored based on a review of at least 3 different pages and no individual page was reviewed more than once. For items in the instrument that refer to a specific page or feature (eg, home page–related items and search function items), the review also included those specific pages or features. As each review progressed, the reviewer revised the scores for previously scored items as needed. In general, reviewers examined about 150 pages on each website.

## Results

Baseline data points for 2015 were calculated for both Healthy People 2020 website quality objectives.

### Information Reliability Baseline

**Figure 1:**
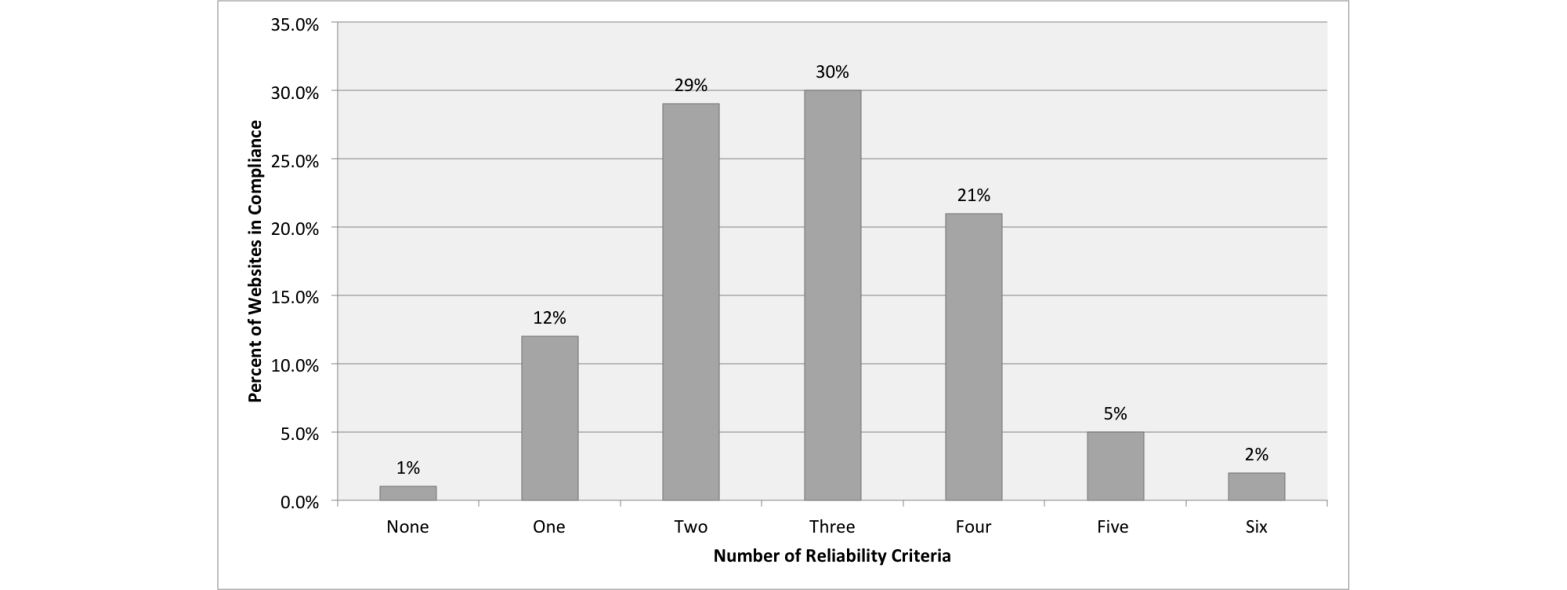
Percentage of websites in compliance, with the number of reliability criteria.

For Healthy People objective HC/HIT-8.1, a total of 58 out of 100 health-related websites (58.0%) met 3 or more of the 6 information reliability criteria. Only 2 out of 100 websites (2.0%) met all the criteria, and 1 out of 100 websites (1.0%) met none of the 6 criteria. See Figure 1.

**Figure 2:**
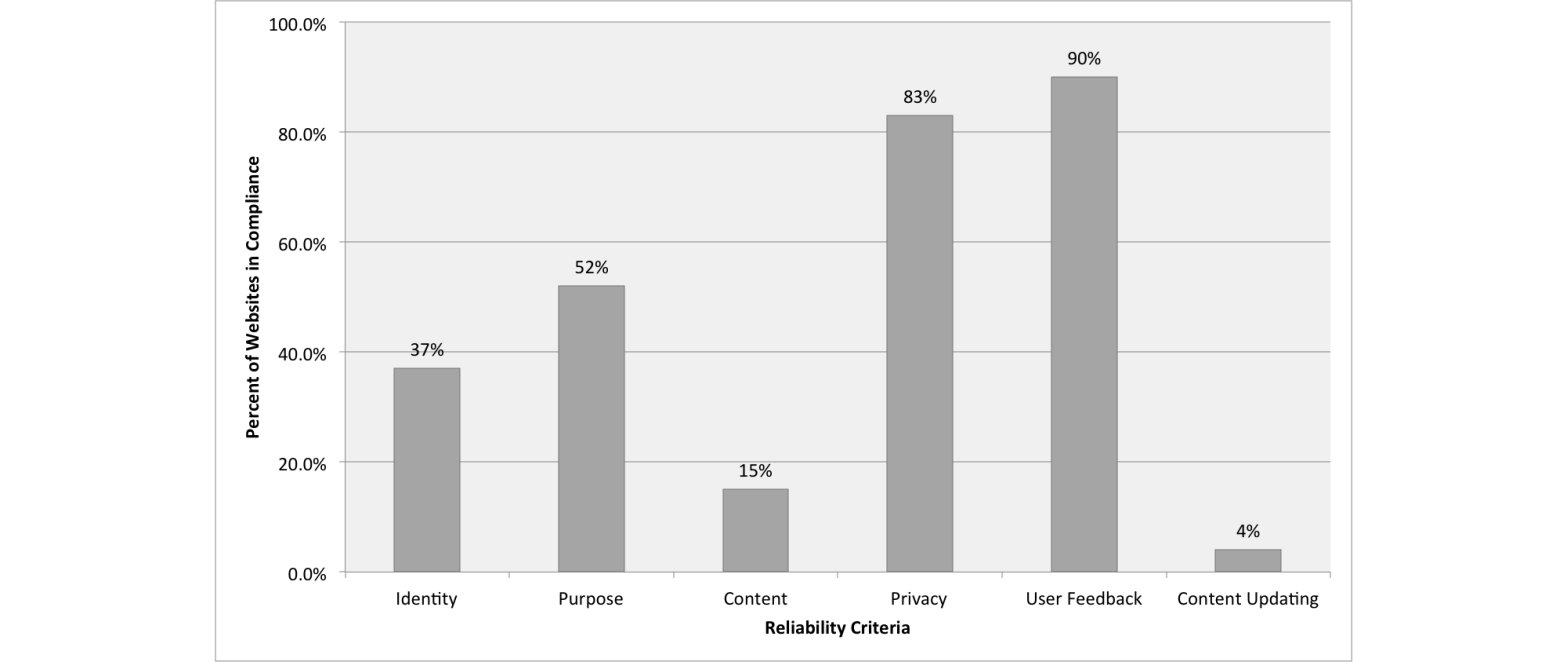
Percentage of websites in compliance, by specific reliability criteria.

Figure 2 shows the percentage of health-related websites in compliance with specific criteria. User Feedback (90.0%) had the highest percentage in compliance, followed by Privacy (83.0%). Only 4 out of 100 websites (4.0%) met the criterion for Content Updating.

Table 4 presents the percentage of websites in compliance by criterion and by information reliability requirements associated with each criterion.

**Table 4:** Estimated percentages of websites in compliance, by information reliability criterion and required disclosure elements.

Criterion and required disclosure elements		N	Count	Percent (%)	SE^a^ (%)	Lower bound 95% CI (%)	Upper bound 95% CI (%)
**Identity**		100	37	37.0	4.83	27.5	46.5
	Name	100	93	93.0	2.55	88.0	98.0
	Street address	100	83	83.0	3.76	75.6	90.4
	Funding sources	100	44	44.0	4.96	34.3	53.7
**Purpose**		100	52	52.0	5.00	42.2	61.8
	Purpose or mission	100	79	79.0	4.07	71.0	87.0
	Uses and limitations	100	82	82.0	3.84	74.5	89.5
	Association with commercial products	100	71	71.0	4.54	62.1	79.9
**Content Development**		100	15	15.0	3.57	8.0	22.0
	Identify advertising content	60	28	46.7	6.44	34.0	59.3
	Describe editorial policy	100	39	39.0	4.88	29.4	48.6
	Authorship	100	38	38.0	4.85	28.5	47.5
**Privacy**		100	83	83.0	3.76	75.6	90.4
	Privacy policy	100	96	96.0	1.96	92.2	99.8
	Describe protection of personal information	100	83	83.0	3.76	75.6	90.4
**User Feedback**		100	90	90.0	3.00	84.1	95.9
	Feedback mechanism	100	90	90.0	3.00	84.1	95.9
**Content Updating**		100	4	4.0	1.96	0.2	7.8
	Display date created	100	25	25.0	4.33	16.5	33.5
	Display date reviewed or updated	100	28	28.0	4.49	19.2	36.8

^a^ SE: standard error.

**Table 5:** Estimated percentages of websites in compliance, by criterion and established usability principles.

Criterion and established usability principles		Number (n=100)	Percent (%)	SE^a^ (%)	Lower bound 95% CI (%)	Upper bound 95% CI (%)
**Site Design**						
	1. Use conventional interaction elements	78	78.0	4.14	69.9	86.1
	2. Make it obvious what is clickable and what is not	68	68.0	4.66	58.9	77.1
	3. Minimize vertical scrolling	24	24.0	4.27	15.6	32.4
	4. Ensure that the Back button behaves predictably	100	100.0	0.00	100.0	100.0
	5. Provide clear feedback signals for actions	39	39.4	4.91	29.8	49.0
	6. Ensure site is accessible for users with disabilities and uses elements of 508 compliance	6	6.0	2.37	1.3	10.7
	7. Provide a simplified user experience	30	30.0	4.58	21.0	39.0
	8. Incorporate multimedia	70	70.0	4.58	61.0	79.0
	9. Offer a functional home page	30	30.0	4.58	21.0	39.0
**Information Architecture**						
	10. Present a clear visual hierarchy	42	42.0	4.94	32.3	51.7
	11. Provide easy search functionality	17	17.0	3.76	9.6	24.4
	12. Clearly label content categories	25	25.0	4.33	16.5	33.5
	13. Make pages easy to skim or scan	45	45.0	4.97	35.2	54.8
	14. Make elements on the page easy to read	72	72.0	4.49	63.2	80.8
	15. Visually group related topics	45	45.0	4.97	35.2	54.8
	16. Make sure text and background colors contrast	74	74.0	4.39	65.4	82.6
**Content Design**						
	17. Focus the writing on audience and purpose	30	30.0	4.58	21.0	39.0
	18. Use the users’ language; minimize jargon and technical terms	32	32.0	4.66	22.9	41.1
	19. Allow for interaction with the content	19	19.0	3.92	11.3	26.7

^a^ SE: standard error.

**Figure 3:**
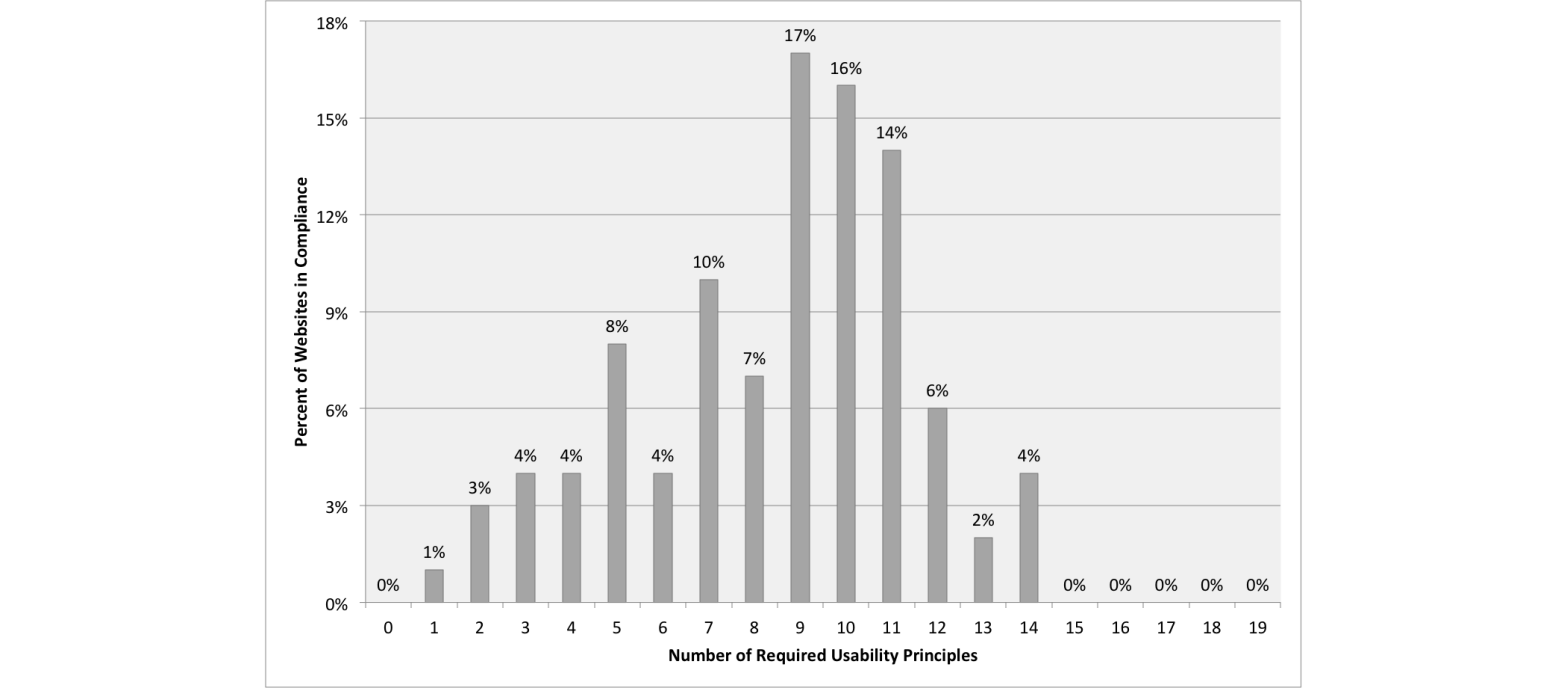
Percentage of websites in compliance, by the number of usability principles.

### Website Usability Baseline

For Healthy People objective HC/HIT-8.2, a total of 42 out of 100 health-related websites (42.0%) met 10 or more out of 19 established usability principles. Figure 3 shows the distribution of website compliance by the number of usability principles met. All websites met at least one usability principle. Almost half of the websites (47.0%) met between 9 and 11 principles. None of the websites met 15 or more of the established usability principles. See Table 5 for the percentage of websites in compliance broken down by the 19 usability principles.

## Discussion

### Baseline Data Points

The 2015 review of health-related websites identified 2 baseline data points. For Healthy People 2020 objective HC/HIT-8.1, a total of 58 out of 100 health-related websites (58.0%) met 3 or more out of 6 reliability criteria. For Healthy People 2020 objective HC/HIT-8.2, a total of 42 out of 100 health-related websites (42.0%) followed 10 or more out of 19 established usability principles.

This research revealed significant shortcomings in the quality of today’s Web-based health information landscape, particularly in disclosing sources of funding and authorship, clearly differentiating between advertisements and original content, complying with universal accessibility guidelines, providing simple search and print functionality, and minimizing scientific and technical jargon.

### 2020 Targets

The ODPHP set the following targets for 2020 that meet the minimal statistical significance. For Healthy People 2020 objective HC/HIT-8.1, 70.5% of health-related websites will meet 3 or more out of 6 reliability criteria. For Healthy People 2020 objective HC/HIT-8.2, 55.7% of health-related websites will follow 10 or more out of 19 established usability principles.

Improving the quality of health-related websites is critical to national efforts to promote health literacy and shared decision making. Until now, there has been no standardized approach to defining and measuring the quality of Web-based health information. With Healthy People 2020 objectives HC/HIT-8.1 and 8.2, ODPHP has developed and validated such an approach, and established baseline data points and national benchmarks to track progress over time.

The 2015 study confirmed that there is, indeed, room for improvement. The ODPHP’s research revealed significant shortcomings in the quality of today’s Web-based health information landscape, particularly in the following areas:

- Disclosing sources of funding and authorship
- Clearly differentiating between advertisements and original content
- Complying with universal accessibility guidelines (eg, Section 508 of the Amendment to the Rehabilitation Act of 1973)
- Providing simple search and print functionality
- Minimizing scientific and technical jargon

### Limitations

This study has several limitations. First, the samples of this study were selected from Alexa Top Sites—health category. The research team had no control over the quality of the website rankings performed by Alexa.

Additionally, some of the usability principles might change over time. For example, in this study, only 24 out of 100 websites (24.0%) followed the principle of minimizing vertical scrolling. However, with the proliferation of mobile and responsive design technology, users are becoming more accustomed to navigating websites by scrolling. Minimizing vertical scrolling may not remain a usability principle in the future.

Finally, the survey instrument is somewhat subjective, especially for objective HC/HIT-8.2. Although the research team controlled the reliability by measuring the IRR for several websites in the sample, there was still variation across reviewers that may affect the results.

### Conclusions

The quality and accessibility of Web-based health information is a key factor in improving access to health services and facilitating informed health decision making. The criteria proposed by ODPHP provide methods to assess the quality of health-related websites and provide baseline data and targets to drive quality improvement. In addition to having implications for website developers and policy makers, this work also points to the need for consumer education related to the quality of Web-based health information. Such education efforts are critical in a time when nearly 3 in 4 Internet users are looking for health information online [42].

To promote increased quality of health-related websites, ODPHP updated and published Health Literacy Online: A Guide to Simplifying the User Experience, second edition [43]. Health Literacy Online is based on literature related to cognitive processing and online behavior and on usability research with more than 800 participants. It features actionable information that website owners, content writers, designers, and developers can use to create quality health websites. The recommendations in Health Literacy Online provide a clear road map for achieving the Healthy People 2020 objectives to increase the proportion of quality health-related websites (Objective HC/HIT-8). The ODPHP is working with other federal agencies to adopt the principles in Health Literacy Online and is presenting these strategies for improving health websites at national conferences.

## Appendix

Multimedia Appendix 1 Website Information Reliability Evaluation Instrument.

Multimedia Appendix 2 Website Usability Evaluation Instrument.

Multimedia Appendix 3 Sample Websites.

## Abbreviations

HC/HIT: Health Communication and Health Information Technology
ICC: interclass correlation
IRR: interrater reliability
ODPHP: Office of Disease Prevention and Health Promotion
