# Supplementary material for: Making Quality Health Websites a National Public Health Priority: Toward Quality Standards
Source: J Med Internet Res. 2016 Aug 2;18(8):e211. doi: 10.2196/jmir.5999 (PMC4987491; doi:10.2196/jmir.5999)
Supplement: Multimedia Appendix 1 [file jmir_v18i8e211_app1.pdf]

Website Information Reliability Evaluation Instrument

Website Name:
Website Homepage URL:
Type of site:
Date Accessed:
Rater:
Coding Start Time:
Coding End Time:

I. IDENTITY

1. Does the website identify by name the person or organization responsible for the website, within two clicks of the homepage?

   __ Yes

   __ No

   Explanation: This is intended to refer to the individual, business, corporation, association, coalition, or group that the user would identify as the website sponsor. Note that responsible entity is distinct from the webmaster or other contractor to whom day-to-day website functions may have been delegated.

2. Does the website provide the following contact information for the person or organization responsible for the website, within two clicks of the homepage?

   __ Street address

   __ Other mailing address (e.g., post office box, mailstop)

   __ Telephone number

   __ Email address

   __ Other contact information [**Optional, not include for scoring]:

3. Does the website provide the following information on sources of funding for the website, within two clicks of the homepage?

   __ Includes explicit statement about sources of funding for website

   __ Names individual or organizational sponsors, donors, or financial partners for

website

Explanation: Note that this refers to funding for the website, not for the sponsoring organization. This information may be found in an advertising or sponsorship policy.

II. PURPOSE

1. Does the website provide information about the purpose or mission of the website, within two clicks of the homepage?

   __ Yes

   __ No

   Explanation: Note that this refers to the purpose or mission of the website, and not of the sponsoring organization. It may include a statement of purpose or a description of services provided to website users such as health information, discussion groups or forums, advice from professionals, support for health services, tools for self management, or the sale of products or services.

2. Does the website describe appropriate uses and limitations of the services it provides, within two clicks of the homepage?

   __ Yes

   __ No

   Explanation: This may include terms and conditions regarding the provision of services, statements that advice or information is not intended to replace the evaluation of a health care professional, statements about the rights and responsibilities of users or chat room participants, or other disclaimers.

3. Does the website include a statement regarding its association with commercial products or services, within two clicks of the homepage?

   __ Yes

   __ No

   Explanation: This may include a statement that the website has no financial interest or association with any product or service mentioned; a statement

disclosing a financial interest or association with a product or service mentioned; or a statement that it endorses no product or service mentioned on the website.

III. CONTENT DEVELOPMENT/EDITORIAL POLICY

1. Does the website clearly differentiate between advertising and non-advertising content?

   __ Yes

   __ No

   Explanation: Look at advertising on the home page and on at least two links from the homepage. Advertising, including sponsored health content, should be clearly distinguished from non-advertising content using identifying words, design, or placement. Answer "yes" to this question only if all advertisements found are clearly marked. "Not applicable" should be selected ONLY if no advertising is found on the site.

2. Does the website describe how it oversees its health content in the following ways, within two clicks of the homepage?

   __ Describes its editorial or medical review process

   Explanation: Note that this should include a description of the process, and not just a statement that content is reviewed.

   __ Provides names and credentials of medical/scientific editors, reviewers, or advisors [**Optional, not include for scoring]

   Explanation: Credentials may include degrees, licensure, titles, academic or clinical affiliations, or areas of professional expertise. If the website provides the names and credentials of medial/scientific advisors, it must clearly state that these advisors oversee health content.

   __ Describes its policy for keeping health content current

   Explanation: Note that this should include a description of the policy, and not just a statement that content is kept current.

   __ Describes other quality oversight practices (explain in comments)

3. Does the website disclose the author of this health-related content in the following ways, within one click of health content?[1]

   __ States that the content is supplied by the website's sponsoring organization or staff

   __ States the name of an organization other than the website sponsor as supplying the content

   __ Identifies individual authors of content by name

   Explanation: When the health content is a .pdf file, it should be considered a stand-alone document. Look for the disclosure items only on the .pdf file. Document the page number where the disclosure item was found.

IV. PRIVACY AND CONFIDENTIALITY

1. Does the website describe its privacy policy, within two clicks of the homepage?

   __ Yes

   __ No

2. Does the site explain how users' personal information is protected, within two clicks of the homepage?

   __ Yes

   __ No

   Explanation: "Personal information" may include email addresses or email exchanges, personal health information, or information derived through the use of passive tracking mechanisms ("cookies").

V. USER FEEDBACK

1. Does the website provide the following specific mechanisms for user feedback about the website, within two clicks of the homepage?

[1] For this question the coder will visit three randomly selected pages of health content that are accessible through direct paths from the website's homepage.

__ Feedback form

Explanation: Feedback form refers to a form that is clearly marked as a means for submitting comments or questions about the website.

__ Pop-up user survey

__ Email address

__ Other feedback mechanism (explain in comments)

2. Does the website describe how it uses information from users to improve its services or operations, within two clicks of the homepage? [**Optional, not include for scoring]

   __ Yes

   __ No

VI. CONTENT UPDATING[2]

1. Does this page of health content display the date this content was created?

   __ Yes

   __ No

   Explanation: The date may be indicated as a year, month and year, or month, day, and year. This question DOES NOT refer to copyright date. If there is a date listed with no other explanation, count this as the date created.

2. Does this page of health content display the date this content was last reviewed and/or updated in the following ways?

   __ Displays date last reviewed or verified

   __ Displays date last updated, modified, or revised

---

[2] For questions 1–3 in this section, the coder will visit three randomly selected pages of health content that are accessible through direct paths from the website's homepage.

Explanation: The date may be indicated as a year, month and year, or month, day, and year.

3. Does this page of health content display a copyright date? [**Optional, not include for scoring]

__ Yes

__ No

Explanation: The date may be indicated as a year, month and year, month, day, and year, or a range of years.
