# Supplementary material for: Making Quality Health Websites a National Public Health Priority: Toward Quality Standards
Source: J Med Internet Res. 2016 Aug 2;18(8):e211. doi: 10.2196/jmir.5999 (PMC4987491; doi:10.2196/jmir.5999)
Supplement: Multimedia Appendix 2 [file jmir_v18i8e211_app2.pdf]

Website Usability Evaluation Instrument

Website Name:
Website Homepage URL:
Type of site:
Date Accessed:
Rater:
Coding Start Time:
Coding End Time:

SCORING

4 = Minimal problems – satisfies the heuristic
3 = Minor hindrance – possible issue, but probably will not hinder this persona/user
2 = Serious problem – may hinder this persona/user
1 = Task failure – prevents this persona/user going further

I. SITE DESIGN

1. *Use conventional interaction elements*

   Are all links clearly indicated in the same manner (such as in the same font, with underlined text)?
   Score: __

   Are links embedded in descriptive text (rather than "click here," or something similar)?
   Score: __

2. *Make it obvious what is clickable and what is not*

   Are clickable items easy to target and hit?
   Score: __

   Are buttons clearly identified, large enough to easily see and click?
   Score: __

   Does the site use text links rather than image links?
   Score: __

3. *Minimize vertical scrolling*

   Does the site use paging (having shorter pages that require to user to frequently move from page to page) rather than scrolling (having longer pages that require scrolling down more than one page to see the end)?

Score: __

Are there visual cues in the layout of the page that help users know there is more content “below the fold”?
Score: __

4. *Ensure that the Back button behaves predictably*

   Is the Back button functional on the browser toolbar on every page?
   Score: __

   Does clicking the Back button always go back to the page from which the user came?
   Score: __

5. *Provide clear feedback signals for actions*

   Are error messages informative and provide solutions to the user?
   Score: __

   Do links and buttons clearly describe what people will find on the next page (using different, distinct, and relevant key words)?
   Score: __

6. *Ensure site is accessible for users with disabilities and uses elements of 508 compliance*

   Is ALT text provided for links, images, video, and animation (this text should pop up when a user hovers the mouse over the element in question)?
   Score: __

   Is captioning provided for video and animation?
   Score: __

   Is such captioning easy to read (in terms of size and contrast)?
   Score: __

7. *Provide a simplified user experience*

   Does the site include print options and printer-friendly tools?
   Score: __

   Does the site provide a feedback mechanism for users (such as comment tools or easily identified contact information)?
   Score: __

Is it easy to get back to the homepage from anywhere in the site with just one click?
Score: __

8. *Incorporate multimedia*

Does the site include audio and visual features?
Score: __

Are images and other multimedia relevant to, and supportive of, the text content?
Score: __

9. *Offer a functional homepage*

Does the homepage look like a homepage (including mostly clear navigation items and general site info, without too much detailed content)?
Score: __

Is the homepage simple yet engaging?
Score: __

Does the homepage state the purpose of the site?
Score: __

Does the homepage enable easy access to navigational items?
Score: __

Is it easy to navigate to the homepage, with one click, from anywhere in the site?
Score: __

II. INFORMATION ARCHITECTURE

1. *Present a clear visual hierarchy*

Is there a clear visual “starting point” to the page?
Score: __

Is the path users took to get to their current page clearly displayed (such as with breadcrumbs)?
Score: __

Are options for next navigational steps clearly displayed (such as with signposting)?
Score: __

Do users find that information is presented with a greater level of detail the further they navigate into the site (i.e., telescoping)?
Score: __

2. *Provide easy search functionality*

Is there a universally located simple option for searching the site?
Score: __

Does the search option provide corrective options (recommendations for misspelled search terms)?
Score: __

Does the search option provide predictive text (auto-filling predicted search terms)?
Score: __

Is there a simple option for browsing the site (such as a directory of all site topics or a navigational structure)?
Score: __

3. *Clearly label content categories*

Are labels descriptive enough to make it easy to accurately predict what the content will be under each topic category?
Score: __

Are labels understandable on their own?
Score: __

4. *Make pages easy to skim or scan*

If pages are dense with content, is content grouped or otherwise clustered to show what is related?
Score: __

Is white space used to break up and clusters of content?
Score: __

Is it easy to tell what is content and what is external advertising?
Score: __

Is it easy to tell what content is part of the page's main body?
Score: __

Do pages use bullets and lists?
Score: __

Are page elements aligned either vertically or horizontally?
Score: __

5. *Make elements on the page easy to read*

Is the default type size at least 12-point?
Score: __

Are headings noticeably larger than body content (between 14-point and 24-point)?
Score: __

Is text set in a type face that is easy to read (without unnecessary flourishes)?
Score: __

Are headings set in a type face that is easy to read (without unnecessary flourishes)?
Score: __

Are there visual cues (such as icons, text boxes, and different colors) to direct users' attention to important items?
Score: __

6. *Visually group related topics*

Are frequently used topics, actions, and links found on the screen without needing to scroll down more than one page?
Score: __

Is important information at the top center of the page?
Score: __

Is there a template applied consistently across the site?
Score: __

7. *Make sure text and background colors contrast*

Do the colors that are used together make information easy to see and find, and have enough contrast to make items easy to read?
Score: __

Are clickable items highlighted differently from other non-clickable highlighted items?
Score: __

III. CONTENT DESIGN

1. *Focus the writing on audience and purpose*

   Is the content written in the active voice, directed to the reader (using "you" as though the page is "talking" to the reader)?
   Score: __

   Are sentences short (20 words or fewer), simple, and straightforward?
   Score: __

   Are paragraphs short and scannable (covering only one subject, and under 12 lines)?
   Score: __

   Are headings, labels, and captions describe the content piece's purpose?
   Score: __

2. *Use the users' language; minimize jargon and technical terms*

   Does the site use mixed case prose (sentences with upper and lower case letters)?
   Score: __

   Does the site use words familiar to the audience (without needing to refer to a dictionary)?
   Score: __

   If there are new or technical terms, does the site help users learn what the terms mean?
   Score: __

   Does the site define acronyms before using them?
   Score: __

3. *Allow for interaction with the content*

   Are users able to input information and preferences that result in tailored content?
   Score: __

   Are users able to share the content with others (do pages include email functions, Facebook, Twitter, or other sharing social media sharing buttons)?
   Score: __
