# Supplementary material for: Making Quality Health Websites a National Public Health Priority: Toward Quality Standards
Source: J Med Internet Res. 2016 Aug 2;18(8):e211. doi: 10.2196/jmir.5999 (PMC4987491; doi:10.2196/jmir.5999)
Supplement: Multimedia Appendix 3 [file jmir_v18i8e211_app3.pdf]

100 Sampled Websites For Evaluation

(Data were pulled on 10-14-2014)

No.	Website	URL	Alexa Rank	Sub-category
1	National Institutes of Health (NIH)	www.nih.gov	1	Government
2	WebMD	www.webmd.com	2	For Profit
3	PubMed	www.ncbi.nlm.nih.gov/pubmed	3	Government
4	MyFitnessPal, Inc.	www.myfitnesspal.com	4	For Profit
5	Mayo Clinic	www.mayoclinic.org	5	Nonprofit
6	Centers for Disease Control and Prevention	www.cdc.gov	6	Government
7	MedlinePlus	www.nlm.nih.gov/medlineplus	7	Government
8	Drugs.com	www.drugs.com	8	For Profit
9	Mercola.com	www.mercola.com	9	For Profit
10	MedicineNet.com	www.medicinenet.com	10	For Profit
11	Psychology Today	www.psychologytoday.com	11	For Profit
12	PMC	www.ncbi.nlm.nih.gov/pmc	12	Government
13	Men’s Health	www.menshealth.com	15	For Profit
14	Health	www.health.com/health	16	For Profit
15	Medscape	www.medscape.com	17	For Profit
16	Fox News Health	www.foxnews.com/health/index.html	18	For Profit
17	Men’s Fitness	www.mensfitness.com	19	For Profit
18	Prevention	www.prevention.com	20	For Profit
19	Kaiser Permanente	www.kaiserpermanente.org	21	Nonprofit
20	Weight Watchers	www.weightwatchers.com	22	For Profit
21	U.S. Food and Drug Administration	www.fda.gov	24	Government
22	RxList	www.rxlist.com	25	For Profit
23	ZocDoc	www.zocdoc.com	26	For Profit
24	SELF	www.self.com	27	For Profit
25	Kids Health	www.kidshealth.org	28	Nonprofit
26	American Cancer Society	www.cancer.org	30	Government
27	Authority Nutrition	www.authoritynutrition.com	31	For Profit
28	Fitness Magazine	www.fitnessmagazine.com	32	For Profit
29	American Psychological Association	www.apa.org	34	Nonprofit
30	Vitals	www.vitals.com	38	For Profit
31	Share Care	www.sharecare.com	40	For Profit
32	Top 10 Home Remedies	www.top10homeremedies.com	42	For Profit

No.	Website	URL	Alexa Rank	Sub-category
33	Spine Health	www.spine-health.com	44	For Profit
34	National Cancer Institute	www.cancer.gov	46	Government
35	Cleveland Clinic	www.clevelandclinic.org	49	Nonprofit
36	American Heart Association	www.heart.org	50	Nonprofit
37	Health Boards	www.healthboards.com	51	For Profit
38	Yahoo Health	www.yahoo.com/health	54	For Profit
39	WEIL Andrew Weil, MD	www.drweil.com	55	For Profit
40	Cure Zone	www.curezone.com	56	For Profit
41	Alzheimer's Association	www.alz.org	58	Nonprofit
42	Johns Hopkins Medicine	www.hopkinsmedicine.org	59	Nonprofit
43	American Diabetes Association	www.diabetes.org	62	Nonprofit
44	Federal Emergency Management Agency	www.fema.gov	65	Government
45	The New England Journal of Medicine	www.nejm.org	68	Nonprofit
46	Sutter Health	www.sutterhealth.org	69	Nonprofit
47	WELLNESS	www.wellness.com	73	For Profit
48	Planned Parenthood	www.plannedparenthood.org	76	Nonprofit
49	ClinicalTrials.gov	www.clinicaltrials.gov	78	Nonprofit
50	Lab Tests Online	www.labtestsonline.org	84	Nonprofit
51	Examine.com	www.examine.com	86	For Profit
52	Calorie King	www.calorieking.com/foods	87	For Profit
53	EARTHCLINIC	www.earthclinic.com	88	For Profit
54	National Institute of Diabetes and Digestive and Kidney Diseases	www.niddk.nih.gov	90	Government
55	Social Anxiety Support	www.socialanxietysupport.com	94	For Profit
56	Fit Pregnancy	www.fitpregnancy.com	95	For Profit
57	The Lancet	www.thelancet.com	100	For Profit
58	Kevin MD	www.kevinmd.com	106	For Profit
59	MD Anderson Cancer Center	www.mdanderson.org	108	Nonprofit
60	GoodTherapy.org	www.goodtherapy.org	110	Nonprofit
61	Science-Based Medicine	www.sciencebasedmedicine.org	111	Nonprofit
62	Additude	www.additudemag.com	114	For Profit
63	Family Doctor	www.familydoctor.org	116	Nonprofit
64	Substance Abuse and Mental Health Services Administration	www.samhsa.gov	118	Nonprofit

No.	Website	URL	Alexa Rank	Sub-category
65	National Institute of Neurological Disorders and Stroke	www.ninds.nih.gov	119	Government
66	CNN Health	www.cnn.com/health	120	For Profit
67	American Society for Nutrition	www.nutrition.org	122	Nonprofit
68	A Place for Mom	www.aplaceformom.com	123	For Profit
69	National Institute on Drug Abuse	www.drugabuse.gov	124	Government
70	ALS Association	www.alsa.org	125	Nonprofit
71	MD Linx	www.mdlinx.com	130	For Profit
72	American Academy of Dermatology	www.aad.org	131	Nonprofit
73	Journal of the American Heart Association	www.circ.ahajournals.org	133	Nonprofit
74	Memorial Sloan Kettering Cancer center	www.mskcc.org	136	Nonprofit
75	Providence Health & Services	www.providence.org	141	Nonprofit
76	DoctorsLounge	www.doctorslounge.com	143	For Profit
77	Agency for Healthcare Research and Quality	www.ahrq.gov	144	Government
78	National Institute of Mental Health	www.nimh.nih.gov	147	Government
79	Breast Cancer.org	www.breastcancer.org	148	Nonprofit
80	National Multiple Sclerosis Society	www.nationalmssociety.org	149	Nonprofit
81	The Rehab Guide	www.therehabguide.com	150	For Profit
82	American Medical Association	www.ama-assn.org	152	Nonprofit
83	Intermountain Healthcare	www.intermountainhealthcare.org	155	Nonprofit
84	Davita	www.davita.com	156	For Profit
85	3FC	www.3fatchicks.com	163	For Profit
86	PXE international	www.pxe.org	164	Nonprofit
87	eMEDtv	www.emedtv.com	166	For Profit
88	Partners Healthcare	www.partners.org	167	Nonprofit
89	National Alliance on Mental Illness	www.nami.org	168	Nonprofit
90	Merck Manuals	www.merckmanuals.com	169	For Profit
91	National Sleep Foundation	www.sleepfoundation.org	170	Nonprofit
92	UCLA Health	www.uclahealth.org	171	Nonprofit
93	St. Jude Children's Research Hospital	www.stjude.org	174	Nonprofit
94	Cancer Treatment Centers of America	www.cancercenter.com	175	For Profit

No.	Website	URL	Alexa Rank	Sub-category
95	National Academy of Sports Medicine	www.nasm.org	176	Nonprofit
96	The New York Times, Health	www.nytimes.com/pages/health	177	For Profit
97	Obesity Help	www.obesityhelp.com	178	For Profit
98	JDRF	www.Jdrf.org	181	Nonprofit
99	Cancer.net	www.cancer.net	182	Nonprofit
100	Wrong Planet	www.wrongplanet.net	183	For Profit
